# TGF‐β induces the differentiation of human CXCL13‐producing CD4^+^ T cells

**DOI:** 10.1002/eji.201546043

**Published:** 2015-11-24

**Authors:** Shio Kobayashi, Takeshi Watanabe, Ryo Suzuki, Moritoshi Furu, Hiromu Ito, Juichi Ito, Shuichi Matsuda, Hiroyuki Yoshitomi

**Affiliations:** ^1^ Center for Innovation in Immunoregulative Technology and TherapeuticsKyoto University Graduate School of MedicineKyotoJapan; ^2^ The Tazuke‐Kofukai Medical Research Institute, Kitano HospitalOsakaJapan; ^3^ Department of OtolaryngologyHead and Neck SurgeryKyoto University Graduate School of MedicineKyotoJapan; ^4^ Department of the Control for Rheumatic DiseasesKyoto University Graduate School of MedicineKyotoJapan; ^5^ Department of Orthopaedic SurgeryKyoto University Graduate School of MedicineKyotoJapan; ^6^ Department of Tissue RegenerationInstitute for Frontier Medical SciencesKyoto UniversityKyotoJapan

**Keywords:** CXCL13, Follicular helper T cells, FoxP3, Proinflammatory cytokines, TGF‐β ⋅ Treg cells

## Abstract

In the ectopic lymphoid‐like structures present in chronic inflammatory conditions such as rheumatoid arthritis, a subset of human effector memory CD4^+^ T cells that lacks features of follicular helper T (Tfh) cells produces CXCL13. Here, we report that TGF‐β induces the differentiation of human CXCL13‐producing CD4^+^ T cells from naïve CD4^+^ T cells. The TGF‐β‐induced CXCL13‐producing CD4^+^ T cells do not express CXCR5, B‐cell lymphoma 6 (BCL6), and other Tfh‐cell markers. Furthermore, expression levels of CD25 (IL‐2Rα) in CXCL13‐producing CD4^+^ T cells are significantly lower than those in FoxP3^+^ in vitro induced Treg cells. Consistent with this, neutralization of IL‐2 and knockdown of STAT5 clearly upregulate CXCL13 production by CD4^+^ T cells, while downregulating the expression of FoxP3. Furthermore, overexpression of FoxP3 in naïve CD4^+^ T cells downregulates CXCL13 production, and knockdown of FoxP3 fails to inhibit the differentiation of CXCL13‐producing CD4^+^ T cells. As reported in rheumatoid arthritis, proinflammatory cytokines enhance secondary CXCL13 production from reactivated CXCL13‐producing CD4^+^ T cells. Our findings demonstrate that CXCL13‐producing CD4^+^ T cells lacking Tfh‐cell features differentiate via TGF‐β signaling but not via FoxP3, and exert their function in IL‐2‐limited but TGF‐β‐rich and proinflammatory cytokine‐rich inflammatory conditions.

## Introduction

Ectopic lymphoid‐like structures (ELSs), discrete clusters of T cells, B cells, macrophages, and dendritic cells (DCs) have been observed in inflamed sites of infections, tumors, and autoimmune diseases 1, 2, 3. These structures support several types of immune responses including antibody production, class switching, and affinity maturation, and the induction of effector functions, leading to protective immunity in infections, better prognosis in cancers, and autoantibody production or treatment‐resistance in autoimmune diseases 1, 3, 4, 5. Levels of chemokines including C‐X‐C motif chemokine 12 (CXCL12), CXCL13, C‐C motif chemokine 19 (CCL19), and CCL21 are elevated in these inflamed tissues and are considered to regulate the cellular distribution and functional features of ELSs 1. Especially, ectopic expression of CXCL13 is sufficient for the generation of ELSs 6. While follicular DCs are well known to contribute to the generation of GC in secondary lymphoid organs via CXCL13 production 7, 8, human CD4^+^ T cells have been noted to produce CXCL13 in ELSs 1, 3, 9, 10, 11, 12.

Human follicular helper T (Tfh) cells produce CXCL13, whereas mouse Tfh cells do not 11, 13, 14, 15. Comprehensive mRNA analysis of human CD4^+^ T cells infiltrating into breast cancers has demonstrated that the expression of Tfh cell signature genes including CXCL13 correlates with better outcomes 3, implying the involvement of CXCL13‐producing Tfh cells in local immune responses of ELSs 1, 11, 12. In contrast, a subset of CD4^+^ T cells that lack typical Tfh cell features also produces CXCL13 in ELSs and is considered to contribute to ELS function 1, 9, 10. In inflamed joints of rheumatoid arthritis (RA), ∼10% of effector memory CD4^+^ alpha/beta T cells produce CXCL13 without the expression of IFN‐γ, IL‐4, IL‐17, or B‐cell lymphoma 6 (BCL6) and express low levels of CXCR5 and ICOS 9, 10. Interestingly, proinflammatory cytokines enhance the production of CXCL13 from synovial CD4^+^ T cells, indicating the influence of local inflammatory conditions on the function of CXCL13‐producing CD4^+^ T cells 10. Consistent with this, blood CD4^+^ T cells of RA patients or healthy controls rarely produce CXCL13 10. In a previous study, we showed that TCR stimulation of blood CD4^+^ T cells induced de novo ∼2% of CXCL13‐producing CD4^+^ T cells, and that proinflammatory cytokines slightly enhanced this differentiation 10. However, these findings did not explain enough how CXCL13‐producing were generated in inflammatory conditions.

Here, we report that TGF‐β induces the differentiation of CXCL13‐producing CD4^+^ T cells that lack Tfh cell like features. We found that TCR stimulation of naïve CD4^+^ T cells with TGF‐β induced CXCL13‐producing CD4^+^ T cells, showing a CXCR5^–^BCL6^–^ICOS^lo^ phenotype and lacking the expression of other Tfh cell signature genes. Interestingly, expression levels of CD25 (IL‐2Rα) in CXCL13‐producing CD4^+^ T cells were significantly lower than those in FoxP3^+^ in vitro induced Treg (iTreg) cells. Consistent with this, attenuation of IL‐2 and STAT5 clearly upregulated CXCL13 expression, and downregulated FoxP3 expression, implying that these expressions were regulated by discrete mechanisms. Furthermore, overexpression of FoxP3 in naïve CD4^+^ T cells downregulated CXCL13 production, and knockdown of FoxP3 failed to inhibit the differentiation of CXCL13‐producing CD4^+^ T cells. These results indicate that the expression of FoxP3 is not required for the induction of TGF‐β‐induced CXCL13‐producing CD4^+^ T cells. As was reported for RA 10, proinflammatory cytokines, together with TGF‐β were crucial for the secondary production of CXCL13 from reactivated CXCL13‐producing CD4^+^ T cells. Thus, TGF‐β induces the differentiation of CXCL13‐producing CD4^+^ T cells that lack typical Tfh cell features, cells that may play a role in the function of ELSs in chronic inflammation.

## Results

### TGF‐β1 induces the differentiation of human CXCL13‐ producing CD4^+^ T cells from naïve CD4^+^ T cells

The cytokine environment during TCR stimulation is critical for the differentiation of effector Th subsets 16. To explore the conditions that induce the generation of CXCL13‐producing cells, we activated human blood CD4^+^ T cells with anti‐CD3/CD28 antibodies under the conditions for differentiation of Th0, Th1, Th2, or Th17. About 3% of blood CD4^+^ T cells produced CXCL13 under Th0 conditions (Supporting Information Fig. 1A), as previously reported 10. Th1 conditions (IL‐12) and Th2 conditions (IL‐4) did not enhance the frequency of CXCL13^+^ cells compared to Th0 conditions (Supporting Information Fig. 1A). Interestingly, a Th17‐polarizing cocktail consisting of TGF‐β1, IL‐1β, and IL‐6 clearly induced the production of CXCL13 (Supporting Information Fig. 1A). Notably, CXCL13 and the relevant cytokine in each differentiation condition showed mutually exclusive pattern (Supporting Information Fig. 1A). To determine the principal factor responsible for the induction of CXCL13‐producing cells, we cultured blood CD4^+^ T cells separately with each cytokine contained in the Th17‐polarizing cocktail. Unexpectedly, TGF‐β1 clearly induced more CXCL13‐producing cells than either IL‐1β or IL‐6 (Supporting Information Fig. 1B). Slight reduction of CXCL13 production in Th17‐polarizing cocktail compared to TGF‐β1 alone supports the possibility that TGF‐β1 is the principal factor responsible for the induction of CXCL13‐producing CD4^+^ T cells.

**Figure 1:**
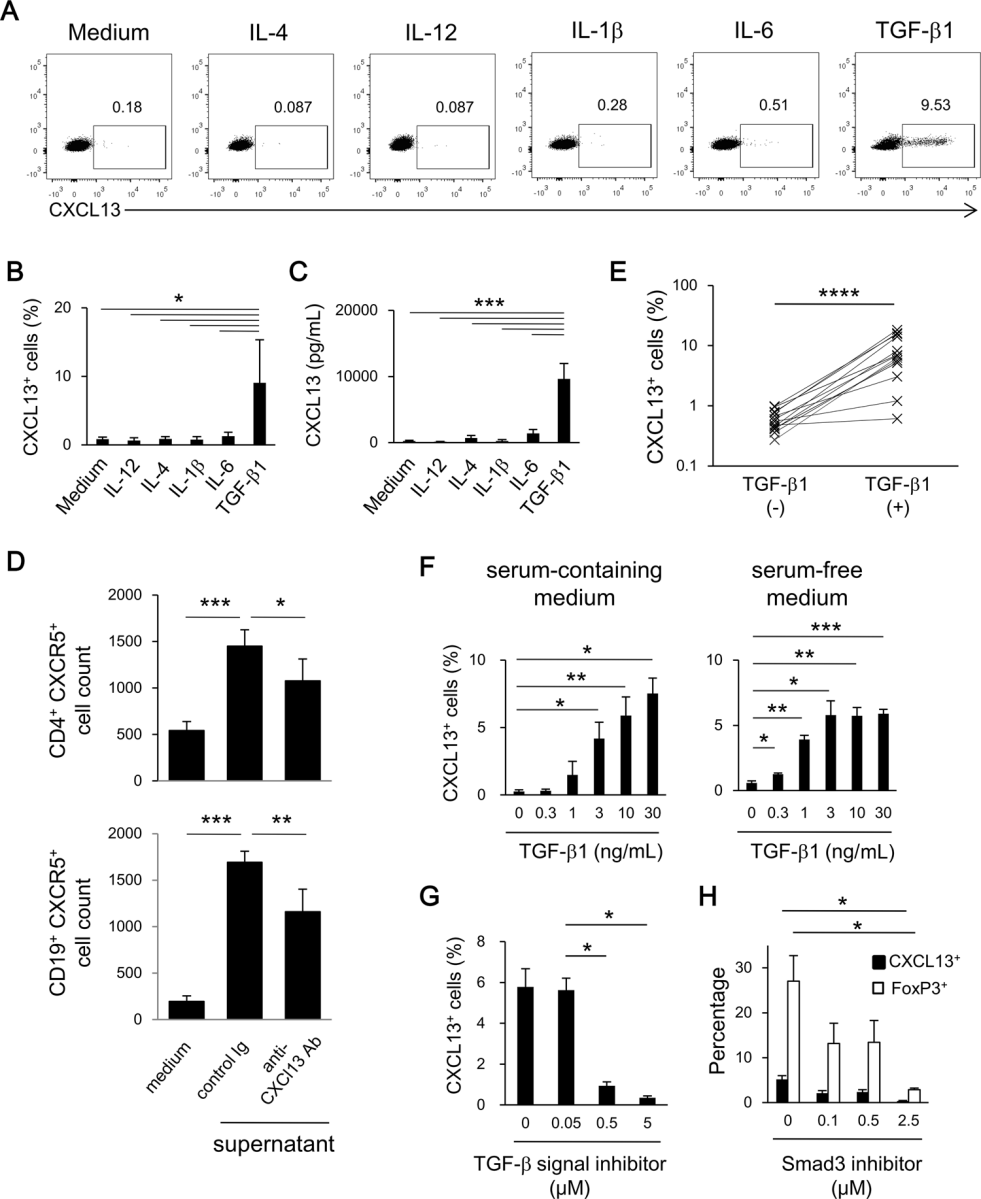
TGF‐β signaling induces the differentiation of CXCL13‐producing CD4^+^ T cells. (A–C) Human naïve blood CD4^+^ T cells were activated in the presence of the indicated cytokine. (A) Representative dot plots, and (B) percentage of CXCL13^+^ cells (*n* = 5) were determined by flow cytometry; and (C) the concentration of CXCL13 in the supernatant on day 7 (*n* = 3) was determined by ELISA. Data are shown as mean + SD of the indicated number of samples from a single experiment representative of three experiments performed. (D) The numbers of CD4^+^CXCR5^+^ (top) and CD19^+^CXCR5^+^ (bottom) cells migrating into medium alone or medium supplemented with 50% supernatant in the presence of the indicated antibody were determined by flow cytometry. The concentration of CXCL13 in the supernatant was 7.8 ng/mL. Data are shown as mean + SD of *n* = 5 samples from one experiment representative of at least three experiments performed. *p < 0.05, **p < 0.01, ***p < 0.001, two‐tailed Student's *t*‐test. (E) Naïve CD4^+^ T cells of 15 healthy volunteers were stimulated with anti‐CD3/28 antibodies in the presence or absence of TGF‐β1. The percentages of CXCL13^+^ cells were determined by flow cytometry; ****p < 0.0001, statistical difference determined by paired Student's *t*‐test. (F) Naïve human blood CD4^+^ T cells were activated in the presence of TGF‐β1 in serum‐containing medium (left) or in serum‐free medium (right). The proportion of CXCL13^+^ cells was determined by flow cytometry. Data are shown as mean + SD of triplicates from a single experiment representative of three experiments performed. (G, H) Naïve blood CD4^+^ T cells were differentiated with TGF‐β1 in the presence of (G) a TGF signal inhibitor, SB431542, or (H) a SMAD3 inhibitor, SIS3. Data are shown as mean + SD of triplicates from a single experiment representative of three experiments performed. Statistical significance determined by two‐tailed Student's *t*‐test.

Next, we investigated whether naïve CD4^+^ T cells were able to differentiate into CXCL13‐producing CD4^+^ T cells. TGF‐β1 induced significantly more CXCL13‐producing cells compared with the other cytokines (Fig. 1A and B). IL‐21 also failed to induce CXCL13 production (data not shown). The concentration of CXCL13 in the culture medium of cells differentiated with TGF‐β1 reached ∼10 ng/mL on day 7 (Fig. 1C), and recruited cells expressing CXCR5, the cognate receptor for CXCL13, in a CXCL13‐dependent manner (Fig. 1D). We further confirmed the induction of CXCL13‐producing CD4^+^ T cells by TGF‐β1 in naïve CD4^+^ T cells of 15 healthy volunteers (Fig. 1E). The activation of naïve CD4^+^ T cells from all the healthy volunteers reproducibly induced ∼8% of CXCL13‐producing cells in the presence of TGF‐β1, implying that the induction of CXCL13‐producing CD4^+^ T cells by TGF‐β is a general human immune phenomenon. Thus, TGF‐β1 induced the differentiation of human CXCL13‐producing CD4^+^ T cells from naïve CD4^+^ T cells.

### TGF‐β signaling is required for the differentiation of CXCL13‐producing CD4^+^ T cells

In human serum containing medium, TGF‐β1 induced the differentiation of CXCL13‐producing CD4^+^ T cells dose‐dependently (Fig. 1F). To eliminate the possibility that the serum in the culture medium contained unknown factors indispensable for this differentiation, we cultured naïve CD4^+^ T cells with TGF‐β1 in serum‐free medium and confirmed a similar dose dependency of the induction (Fig. 1F), indicating that TGF‐β1 was sufficient for the induction of CXCL13‐producing CD4^+^ T cells. SB431542, an inhibitor of TGF‐β signaling leading to SMAD2/3/4 pathway but not of bone morphogenetic protein (BMP) signaling, suppressed the differentiation of CXCL13‐producing cells (Fig. 1G). Consistent with this, TGF‐β2 and TGF‐β3 induced the differentiation of CXCL13‐producing CD4^+^ T cells to the same degree as TGF‐β1, whereas BMP2 and BMP4 failed to induce their differentiation (Supporting Information Fig. 2). Furthermore, an inhibitor of SMAD3, a transcription factor crucial for the induction of FoxP3 in a TGF‐β‐containing environment 17, significantly inhibited the differentiation of CXCL13‐producing CD4^+^ T cells (Fig. 1H). These results collectively indicate that TGF‐β signaling is required for the differentiation of CXCL13‐producing CD4^+^ T cells from naïve CD4^+^ T cells.

### TGF‐β‐induced CXCL13‐producing CD4^+^ T cells lack features of Tfh cells

Next, we investigated the ability for Th cytokine production of CXCL13‐producing CD4^+^ T cells. TGF‐β‐induced CXCL13‐producing CD4^+^ T cells failed to produce IFN‐γ, IL‐4, or IL‐17 (Supporting Information Fig. 3A), and there were significantly fewer cytokine/CXCL13 double‐positive CD4^+^ T cells than CXCL13 single‐positive cells (Supporting Information Fig. 3B). These results imply that TGF‐β‐induced CXCL13‐producing CD4^+^ T cells are not committed to either Th1, Th2, or Th17 cells.

**Figure 2:**
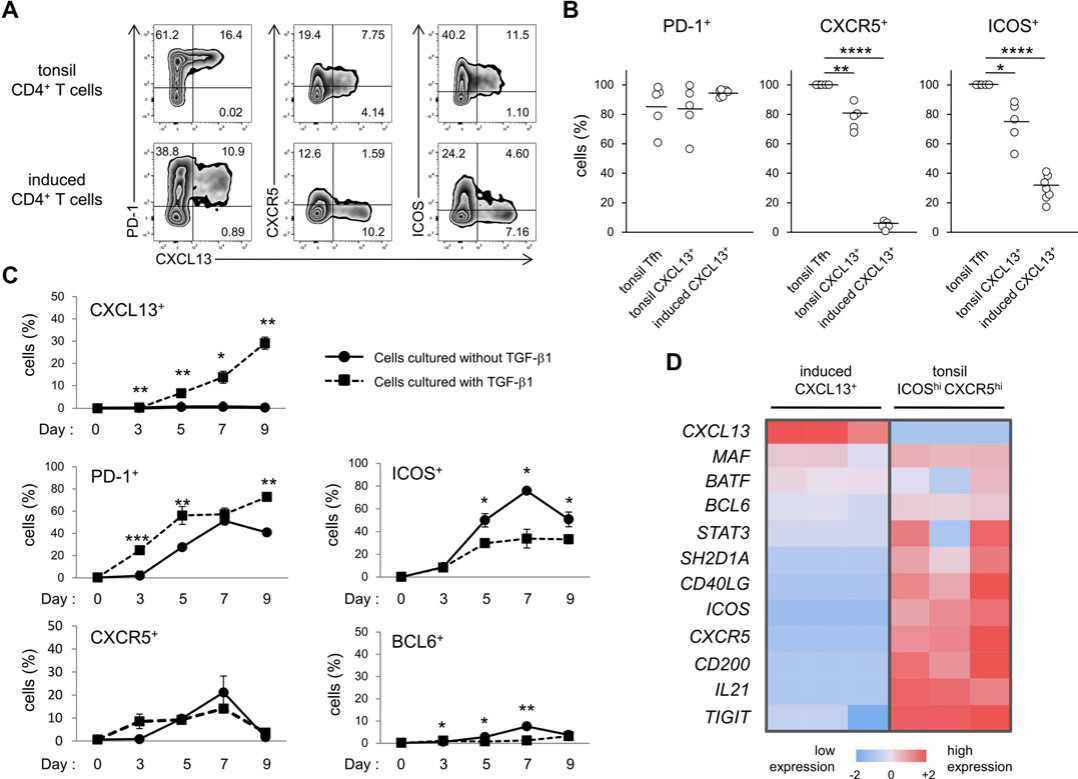
Expression profiles of Tfh‐cell features in TGF‐β‐induced CXCL13‐producing CD4^+^ T cells. (A and B) Expression of PD‐1, CXCR5, ICOS, and CXCL13 on tonsil CD4^+^ T cells (top, *n* = 5) and TGF‐β‐induced CXCL13‐producing CD4^+^ T cells (bottom, *n* = 8) was determined by flow cytometry. (A) Representative dot plots and (B) summaries of tonsil CD3^+^CD4^+^CXCR5^hi^ICOS^hi^ Tfh cells, tonsil CD3^+^CD4^+^CXCL13^+^ cells, and CXCL13‐producing CD4^+^ T cells induced from naïve CD4^+^ T cells are shown. The border of the quadrants was determined according to the staining with isotype controls. Numbers in plots indicate the percentage of cells in each area. Each symbol represents an individual sample and bars represent means. (C) The percentages of CXCL13^+^, PD‐1^+^, ICOS^+^, CXCR5^+^, and BCL6^+^ cells in naïve CD4^+^ T cells differentiated with or without TGF‐β1 on the indicated day were determined by flow cytometry. Data are shown as mean ± SD of triplicate samples from one experiment from three experiments. *p < 0.05, **p < 0.01, ****p < 0.0001, two‐tailed Student's *t*‐test. (D) The heat map of 12 Tfh signature genes in sorted TGF‐β‐induced CXCL13‐producing CD4^+^ T cells and tonsil Tfh cells, as determined by quantitative PCR. Log mRNA expression is shown. Red and blue represent high and low mRNA expression, respectively.

Human Tfh cells, which express programmed death 1 (PD‐1), CXCR5, ICOS, and transcription factor BCL6, are known to produce CXCL13 12, 13, 14, 15, 18. However, a subset of human ELS effector memory Th cells that lacks typical Tfh cell like features also produces CXCL13 1, 9, 10. Therefore, we investigated whether TGF‐β‐induced CXCL13‐producing CD4^+^ T cells have Tfh cell like features. By the flow cytometry, TGF‐β‐induced CXCL13‐producing CD4^+^ T cells were positive for PD‐1, but expressed low levels of CXCR5 and ICOS, whereas tonsil Tfh cells or tonsil CXCL13‐producing CD4^+^ T cells express high levels of PD‐1, CXCR5, and ICOS (Fig. 2A and B). To investigate the involvement of TGF‐β in the induction of Tfh markers, we analyzed the time‐course expression of CXCL13, PD‐1, CXCR5, ICOS, and BCL6. CD4^+^ T cells differentiated with TGF‐β1 started to produce CXCL13 on day 3 (0.29% in TGF‐β condition). In the presence of TGF‐β, the expression of PD‐1 was significantly higher than that in non‐TGF‐β condition, whereas ICOS expression was significantly lower, implying that the upregulation of PD‐1 and the downregulation of ICOS were partly attributed to the presence of TGF‐β1. The expression of CXCR5 in TGF‐β group was constantly ∼10%, whereas CXCR5 expression in the absence of TGF‐β continued to increase until day 7 (Fig. 2C). The expression of BCL6 under the TGF‐β condition remained less than 3% and was significantly lower than that in non‐TGF‐β condition on day 7, implying that the involvement of BCL6 in the induction of CXC13 expression under TGF‐β condition was not likely. Thus, under TGF‐β condition, the expression of major Tfh cell signatures, CXCR5, IOCS, and BCL6 except for PD‐1, was constantly low and did not show temporary upregulation in early or late stage of the differentiation. Consistent with these findings, the quantitative PCR analysis of the expression of other Tfh cell signature genes including *BCL6*, *SH2D1A*, *CD40LG*, *CD200*, *IL21*, and *TIGIT* also demonstrated that TGF‐β‐induced CXCL13‐producing CD4^+^ T cells lack most Tfh cell‐like features (Fig. 2D). Thus, TGF‐β induces the CXCL13‐producing CD4^+^ T cells that lack Tfh cell signatures.

### TGF‐β‐induced CXCL13‐producing CD4^+^ T cells express lower levels of CD25 than FoxP3^+^ iTreg cells

**Figure 3:**
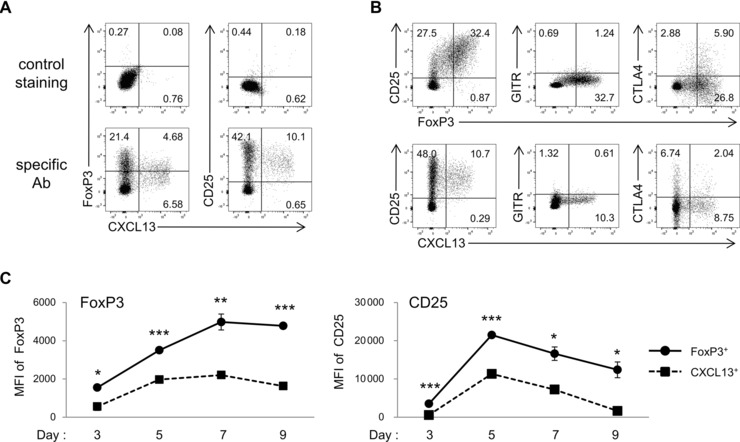
Expression of Treg‐cell markers in TGF‐β‐induced CXCL13‐producing CD4^+^ T cells. (A–C) Naïve CD4^+^ T cells were differentiated with TGF‐β1. (A) The expression of FoxP3 and CD25 in CXCL13^+^ cells, and (B) the expression of CD25, GITR, and CTLA4 in FoxP3^+^ cells or CXCL13^+^ cells were determined by flow cytometry. The border of the quadrant was determined according to the staining with isotype controls. (C) MFI of FoxP3 (left) and CD25 (right) in cells positive for FoxP3 or CXCL13 on the indicated day was determined by flow cytometry. Data are shown as mean ± SD of triplicate samples from one experiment representative of three experiments performed. *p < 0.05, **p < 0.01, ***p < 0.001, two‐tailed Student's *t*‐test. Numbers in plots indicate the percentage of cells in each area.

The requirement of TGF‐β, the crucial cytokine for the generation of FoxP3^+^ iTreg cells 19, 20, in the differentiation of CXCL13‐producing CD4^+^ T cells prompted us to investigate the expression of Treg markers in these cells. The expression of FoxP3 and CD25 in CXCL13^+^ cells was lower than in CXCL13^−^ cells (Fig. 3A). Although the frequencies of glucocorticoid‐induced TNF receptor‐related protein (GITR) or cytotoxic T‐lymphocyte‐associated antigen 4 (CTLA4) were similar between FoxP3^+^ and CXCL13^+^ cells, the expression of CD25 was linearly correlated with the expression of FoxP3, but mutually exclusive with the expression of CXCL13 (Fig. 3B). TGF‐β‐induced CXCL13^+^CD4^+^ T cells differentiated in serum‐free medium showed similar tendency except for lower FoxP3 expression (Supporting Information Fig. 4A and B). The time‐course analysis for FoxP3 and CD25 expression showed that the expression levels of FoxP3 or CD25 in CXCL13^+^ cells were significantly lower than those in FoxP3^+^ cells (Fig. 3C). The expression levels of FoxP3 continued to increase in FoxP3^+^ cells until day 7, whereas that reached almost plateau on day 5 in CXCL13^+^ cells. The expression of CD25 on CXCL13^+^ cells reached a peak on day 5 and almost disappeared on day 9, implying that the temporary CD25 upregulation in CXCL13‐producing CD4^+^ T cells was partly attributed to cell activation. Thus, CXCL13^+^ cells express lower levels of CD25 compared to FoxP3^+^ iTreg cells.

### Attenuation of IL‐2 signaling enhances the differentiation of CXCL13‐producing CD4^+^ T cells

**Figure 4:**
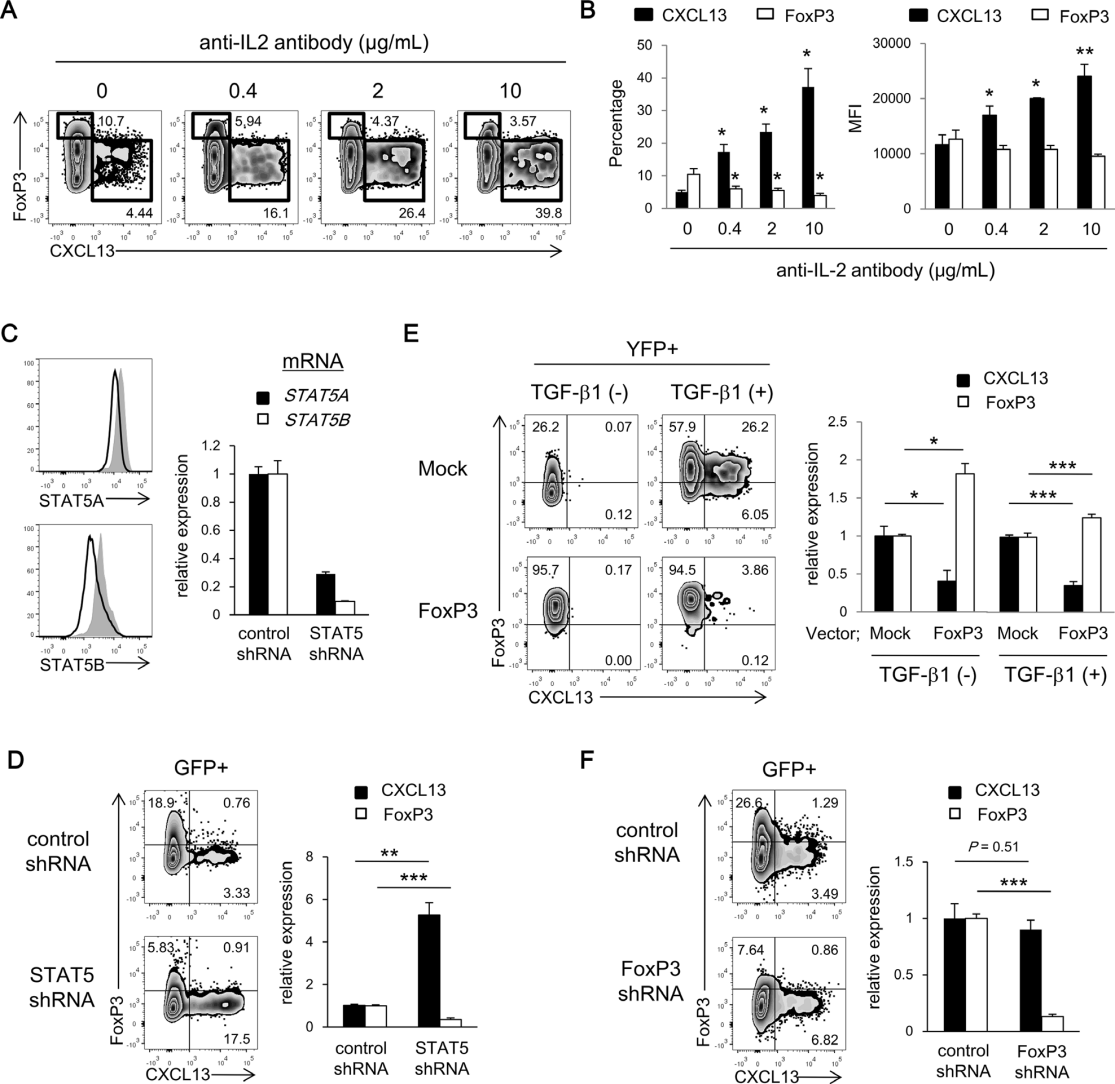
Involvement of the IL‐2 signaling and FoxP3 expression in the differentiation of CXCL13‐producing CD4^+^ T cells. (A and B) Naïve CD4^+^ T cells were differentiated with TGF‐β1 in the presence of neutralizing anti‐IL‐2 antibody. (A) Representative dot plots, (B) frequencies of CXCL13^+^ or FoxP3^hi^ cells (left), and MFI of CXCL13 or FoxP3 in cells positive for CXCL13 or FoxP3, respectively (right), were determined with flow cytometry. Statistical difference between anti‐IL‐2 treatment and the control are shown. *p < 0.05, **p < 0.01, ***p < 0.001, two‐tailed Student's *t*‐test. (C and D) Naïve CD4^+^ T cells transduced with control or STAT5‐specific shRNA by GFP‐expressing lentivirus were differentiated in the presence of TGF‐β1. The shRNA for STAT5 targeted both STAT5A and STAT5B. (C) The expression of STAT5A and STAT5B in GFP^+^ cells was measured by flow cytometry (left) and quantitative PCR (right). Shaded and solid histograms represent control and STAT5‐specific shRNA, respectively. (D) Representative dot plots of GFP^+^ cells (left) and the ratio of CXCL13 or FoxP3 induction in GFP^+^ cells to that in GFP^−^ cells (right) were determined with flow cytometry. (E) Naïve CD4^+^ T cells transduced with mock or FoxP3 by YFP‐expressing (where YFP is yellow fluorescent protein) lentivirus were differentiated with or without TGF‐β1. Representative dot plots of YFP^+^ cells (left) and the ratio of CXCL13 or FoxP3 induction in YFP^+^ cells to that in YFP^−^ cells (right) were determined with flow cytometry. (F) Naïve CD4^+^ T cells transduced with control or FoxP3‐specific shRNA by GFP‐expressing lentivirus were differentiated in the presence of TGF‐β1. Representative dot plots of GFP^+^ cells (left) and the ratio of CXCL13 or FoxP3 induction in GFP^+^ cells to that in GFP^−^ cells (right) were determined with flow cytometry. (B–F) Data are shown as mean + SD of triplicates from a single experiment representative of three experiments performed with statistical difference determined by two‐tailed Student's *t*‐test. Numbers in plots indicate the percentage of cells in each area.

The lower expression of CD25 (IL‐2Rα) in CXCL13‐producing CD4^+^ T cells prompted us to investigate whether IL‐2 signaling, crucial for the differentiation of iTreg cells 21 and the survival of Treg cells 22, was involved in the differentiation of CXCL13‐producing CD4^+^ T cells. Neutralization of IL‐2 clearly upregulated the differentiation of CXCL13‐producing CD4^+^ T cells and downregulated the expression of FoxP3 in a dose‐dependent manner (Fig. 4A and B). Notably, ∼40% of cells differentiated in the presence of anti‐IL‐2 antibody produced CXCL13. These results indicate that endogenous IL‐2 inhibited the differentiation of CXCL13‐producing CD4^+^ T cells, but induced the expression of FoxP3. Next, we knocked down crucial downstream transcription factors of IL‐2 signaling, STAT5A and STAT5B, in naïve CD4^+^ T cells (Fig. 4C), and differentiated CXCL13‐producing CD4^+^ T cells in the presence of TGF‐β1. Consistent with the results for IL‐2 neutralization, knockdown of STAT5 clearly upregulated the differentiation of CXCL13‐producing cells and downregulated the expression of FoxP3 (Fig. 4D). Thus, FoxP3 expression and CXCL13 production are regulated by discrete mechanisms, and CXCL13‐producing CD4^+^ T cells may preferentially differentiate in an IL‐2‐limited environment, such as the RA synovium, where the levels of T cell derived cytokines are relatively low compared with those of proinflammatory cytokines [23, 24].

### FoxP3 is not required for the differentiation of TGF‐β‐induced CXCL13‐producing CD4^+^ T cells

Although the previous results implied that FoxP3 expression and CXCL13 production were regulated by discrete mechanisms, almost a half of TGF‐β‐induced CXCL13‐producing CD4^+^ T cells expressed middle levels of FoxP3 during the differentiation (Fig 3A). To investigate further the involvement of FoxP3 in the differentiation of CXCL13‐producing CD4^+^ T cells, we overexpressed or knocked down FoxP3 in naïve CD4^+^ T cells and differentiated CXCL13‐producing CD4^+^ T cells. Transduction of FoxP3 inhibited the induction of CXCL13 both in the presence and absence of TGF‐β1 (Fig. 4E). Additionally, knockdown of FoxP3 failed to inhibit the differentiation of CXCL13‐producing CD4^+^ T cells in the presence of TGF‐β1 (Fig. 4F). Collectively, these results indicate that FoxP3 function is not required for the induction of CXCL13 production despite middle levels of FoxP3 expression in CXCL13‐producing CD4^+^ T cells.

### Proinflammatory cytokines are crucial for secondary CXCL13 production by TGF‐β‐induced CD4^+^ T cells

**Figure 5:**
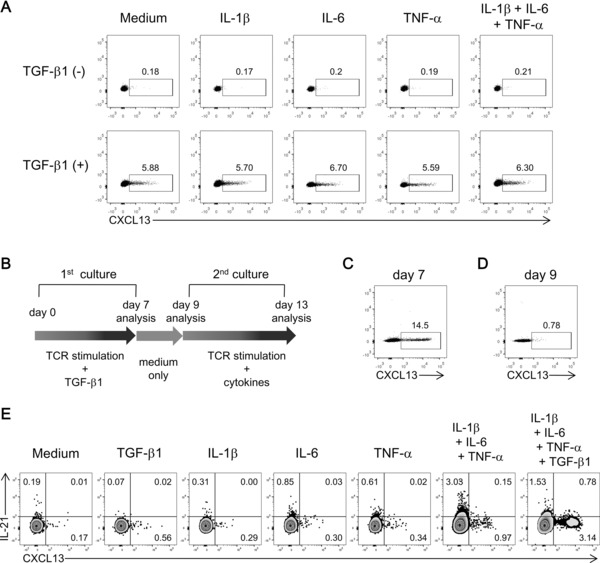
Role of proinflammatory cytokines in the induction of CXCL13‐producing CD4^+^ T cells and in secondary production of CXCL13 by restimulated cells. (A) Naïve CD4^+^ T cells were activated in the presence of IL‐6, TNF‐α, IL‐1β, and/or TGF‐β1. The percentages of CXCL13^+^ cells were determined with flow cytometry. Dot plots from a single experiment representative of three experiments performed were shown. (B–E) Naïve CD4^+^ T cells were activated in the presence of TGF‐β1 for 7 days (C), rested in basal medium for 2 days (D), and restimulated with anti‐CD3/28 antibodies in the presence of TGF‐β1, IL‐1β, IL‐6, and/or TNF‐α for 4 days, followed by intracellular staining after 5 h activation with PMA/ionomycin (E). The percentages of CXCL13^+^ or IL‐21^+^ cells were determined with flow cytometry. Dot plots from a single experiment representative of three experiments performed were shown. Numbers in plots indicate the percentage of cells in each area.

We previously showed that in RA, inflammatory cytokines enhance both the production of CXCL13 by synovial CXCL13‐producing CD4^+^ T cells and the induction of CXCL13‐producing cells from blood CD4^+^ T cells 10. To investigate the effect of proinflammatory cytokines on the differentiation of CXCL13‐producing CD4^+^ T cells, naïve CD4^+^ T cells were cultured in the presence of IL‐6, TNF‐α, and/or IL‐1β with or without TGF‐β1. In the absence of TGF‐β, proinflammatory cytokines including TNF‐α failed to induce the differentiation of CXCL13‐producing CD4^+^ T cells from naïve CD4^+^ T cells. In the presence of TGF‐β, proinflammatory cytokines did not show significant enhancement of the differentiation for CXCL13‐producing CD4^+^ T cells (Fig. 5A), while IL‐6 inhibited the induction of FoxP3 (Supporting Information Fig. 5) as previously reported 25.

Next, we addressed the effect of the presence of proinflammatory cytokines during the TCR‐mediated restimulation of TGF‐β‐induced CXCL13‐producing CD4^+^ T cells. TGF‐β‐induced CXCL13‐producing cells were harvested on day 7, rested in the basal culture medium for 2 days, and then restimulated in the presence of TGF‐β1, IL‐1β, IL‐6, and/or TNF‐α (Fig. 5B to E). Interestingly, the combination of proinflammatory cytokines IL‐6, TNF‐α, and IL‐1β induced the secondary production of CXCL13 from restimulated CD4^+^ T cells (Fig. 5E). In the absence of TCR stimulation, proinflammatory cytokines alone failed to induce the secondary production (data not shown). Addition of exogenous TGF‐β1 to the combination of proinflammatory cytokines enhanced CXCL13 production (Fig. 5E). The expression of IFN‐γ and IL‐17 by restimulated cells under each condition was slight (Supporting Information Fig. 6). The expression of IL‐21 was induced in the presence of IL‐6 as previously reported 26, and upregulated more in the combination of proinflammatory cytokines, whereas downregulated with the addition of exogenous TGF‐β to the combination (Fig 5E). However, the expression of IL‐21 and CXCL13 showed mutually exclusive pattern. Thus, TGF‐β was the factor responsible for the differentiation of CXCL13‐producing cells from naïve CD4^+^ T cells, and proinflammatory cytokines and TGF‐β regulated the secondary production of CXCL13 from reactivated cells. Taken together, our findings demonstrate that in a chronic inflammatory environment such as RA, CXCL13‐producing CD4^+^ T cells that lack Tfh cell‐like features differentiate via TGF‐β signaling, but not via FoxP3, and exert their function, that is, CXCL13 production, especially in inflammatory conditions.

## Discussion

CXCL13 is a chemokine that is crucial for the GC formation in secondary lymphoid organs 7, and ectopic expression of CXCL13 is sufficient for the generation of ELSs 6. The ELSs in chronic inflammatory conditions like active RA synovium contain a considerable number of CXCL13‐producing CD4^+^ T cells that lack typical Tfh cell like features and may contribute to the function of ELSs 1, 9, 10. In this study, we have shown that TGF‐β induces the differentiation of human CXCL13‐producing CD4^+^ T cells that lack Tfh cell features. Although TGF‐β plays a crucial role in the differentiation of iTreg cells via the induction of FoxP3, FoxP3 did not induce CXCL13 production in CD4^+^ T cells. Furthermore, proinflammatory cytokines played a crucial role in the secondary production of CXCL13 by reactivated CXCL13‐producing CD4^+^ T cells. Thus, TGF‐β induces CXCL13‐producing CD4^+^ T cells that lack Tfh cell like features in a FoxP3‐independent manner.

ELSs are frequently present in chronic inflammatory conditions including RA, psoriasis, multiple sclerosis, inflammatory bowel diseases, and tumors, and are considered to play roles in their pathogenesis 1, 2, 3, 27, 28, 29]. In these ELSs, several immune responses including the expression of activation‐induced cytidine deaminase (AID), local production of antibodies or autoantibodies, and induction of effector functions have been reported 1, 5, 30, 31, and further investigation of immune responses related to ELS function is required. In various chronic inflammatory conditions, the expression of CXCL13 in local inflammatory sites is elevated 1 and correlates with disease activity 32, 33, implying the involvement of CXCL13 in the function of ELSs in inflammatory conditions. While follicular DCs are the main source of CXCL13 in secondary lymphoid organs 8, other cells such as murine medial smooth muscle cells 34 and human CD4^+^ T cells including Tfh cells and non‐Tfh memory cells 1, 3, 9, 10 contribute to CXCL13 production in ELSs. However, it has not been determined how these cells, especially non‐Tfh CXCL13‐producing CD4^+^ T cells, are generated.

In chronic inflammatory conditions, resident fibroblasts and macrophages, and infiltrating Treg cells locally produce TGF‐β 35. Acidic conditions in inflamed sites and the presence of reactive oxygen species may be involved in the activation of TGF‐β 36. The involvement of Th17 cells in the pathogenesis of chronic inflammatory diseases 37, 38, 39, 40 supports the presence of local TGF‐β, a factor required for the differentiation of Th17 cells. Thus, TGF‐β in inflammatory sites may contribute to the differentiation of CXCL13‐producing CD4^+^ T cells. In addition to Th17 cells, which are considered to support the generation of ELSs 1, CXCL13‐producing CD4^+^ T cells also partly contribute to the pathogenesis via formation and maintenance of ELSs.

TGF‐β induces the expression of transcription factors FoxP3 19 and RORγt. In this manuscript, we showed that TGF‐β signaling reproducibly induces the differentiation of CXCL13‐producing CD4^+^ T cells. Although in the differentiation of Th17 cells, addition of proinflammatory cytokines is required to overcome the antagonistic effect of TGF‐β toward the function of RORγt 41, TGF‐β is sufficient for the initial induction of CXCL13‐producing CD4^+^ T cells from naïve CD4^+^ T cells. Proinflammatory cytokines IL‐1β, IL‐6, and TNF‐α, together with TGF‐β, induce the secondary production of CXCL13 by reactivated CXCL13‐producing CD4^+^ T cells. In contrast, the function of Treg cells and the differentiation of iTreg cells are abrogated by the proinflammatory cytokine IL‐6 42, 43.

In the presence of TGF‐β, the expression of FoxP3 and CXCL13 was simultaneously upregulated in naïve CD4^+^ T cells. In our study, about a half of TGF‐β‐induced CXCL13‐producing CD4^+^ T cells expressed from low to middle levels of FoxP3. However, neutralization of IL‐2 and knockdown of STAT5 dramatically enhanced the CXCL13 production, but downregulated FoxP3 expression. Furthermore, overexpression or knockdown of FoxP3 showed that FoxP3 does not induce the CXCL13 production by CD4^+^ T cells. Thus, in TGF‐β‐existing inflammatory conditions, a part of FoxP3^low‐middle^ cells, in addition to a part of FoxP3^−^ cells, may produce CXCL13 via TGF‐β signaling but not via FoxP3 activity. IL‐2/STAT5 signaling also constrains murine Th17 differentiation 44. Together with our result with proinflammatory cytokines, these data suggest that in an IL‐2‐limited but TGF‐β‐rich and proinflammatory cytokine‐rich environment such as inflamed joints of RA 23, 24, CXCL13‐producing CD4^+^ T cells and Th17 cells, rather than FoxP3^hi^ cells, may preferentially differentiate and exert their function.

TGF‐β‐induced CXCL13‐producing CD4^+^ T cells are similar to Tfh cells in their expression of PD‐1 18 and CXCL13 13. However, these cells lack most Tfh cell features including BCL6 and CXCR5 expression, and TGF‐β‐induced CXCL13‐producing CD4^+^ T cells differentiate via a simple process, whereas the differentiation of Tfh cells requires sequential subtle processes including induction of CXCR5 expression, priming by DCs, and interaction with B cells 11, 45, 46, 47. Furthermore, the poor expression of Tfh cell signatures in TGF‐β‐induced CXCL13‐producing CD4^+^ T cells imply their low B helper activity. Instead, TGF‐β‐induced non‐Tfh CXCL13‐producing CD4^+^ T cells may differentiate easily under TGF‐β‐rich inflammatory conditions. Locally differentiated TGF‐β‐induced CXCL13‐producing CD4^+^ T cells may function in the recruitment of CXCR5^+^ cells, including circulating Tfh cells 48 and B cells, especially under IL‐2‐limited inflammatory conditions, and play a crucial role in the generation and the maintenance of ELSs, and therefore could be termed inflammatory CXCL13‐producing helper T cells.

Here, we have demonstrated that TGF‐β is required for the differentiation of CXCL13‐producing CD4^+^ T cells that lack Tfh cell features. We cannot confirm reports about remarkable CXCL13 production by murine CD4^+^ T cells 15, reflecting the difficulty of using murine models in the analysis of ELS CXCL13‐producing CD4^+^ T cells. In this context, further clarification of the molecular mechanisms regulating human non‐Tfh CXCL13‐producing CD4^+^ T cells, including the responsible transcription factors downstream of TGF‐β signaling, specific surface molecules, accompanying effector factors, and functions of these cells, may improve our understanding of ELS function and may suggest alternative treatments for chronic inflammatory diseases.

## Materials and methods

### Preparation of specimens

Ethical approval for this study was granted by the ethics committee of Kyoto University Graduate School and Faculty of Medicine. Written informed consent was obtained from all study participants. Tonsils were obtained from five patients (consisting of one female and four male patients ranging from 5 to 48 years old) having a tonsillectomy because of tonsillar hypertrophy. Tonsils were minced and digested with 2.5 mg/mL collagenase D (Roche) at 37°C for 1.5 h and then subsequently analyzed. These studies were exploratory research studies.

Fresh PBMCs from healthy volunteers were collected using Lymphocyte Separation Solution 1.077 (Nacalai Tesque) followed by cell separation and cell culture without cryopreservation. The age of healthy volunteers (consisting of five females and ten males) was ranging from 34 to 54 years old. Cell number was determined with counting chamber (TGK). The average yield of PBMC was 30 million cells per 10 mL peripheral blood with a viability of more than 95%. Naive blood CD4^+^ T cells were purified with naïve CD4^+^ T Cell isolation kit II (Miltenyi Biotec) through column twice as a fraction negative for CD8, CD14, CD15, CD16, CD19, CD25, CD34, CD36, CD45RO, CD56, CD123, TCRγ/δ, HLA‐DR, or CD235a and the purity of CD3^+^CD4^+^CD45RA^+^ cells in the sorted cells was more than 98%.

### Cell culture

Cells were cultured in a humidified, 5% CO_2_ incubator at 37°C with IMDM (Life Technologies) supplemented with 3.5% human AB serum (Lonza), and 100 unit/mL penicillin and streptomycin (Life Technologies) or with serum‐free X‐VIVO™ 15 medium (Lonza). Human AB serum was pretested about the differentiation of human T cells. We investigated the concentration of anti‐CD28 antibodies (CD28.2, eBioscience) optimal for the differentiation of CXCL13‐producing CD4^+^ T cells from naïve CD4^+^ T cells of each healthy volunteer. Unless otherwise indicated, naïve blood CD4^+^ T cells were stimulated with 5 μg/mL plate‐bound anti‐CD3 (OKT3, eBioscience) and 2.5–10 μg/mL soluble anti‐CD28 antibodies in the presence of 5 μg/mL anti‐IFN‐γ (NIB42, BioLegend) and 5 μg/mL anti‐IL‐4 (MP4‐25D2, BioLegend) antibodies, and the indicated cytokines at the concentration of 10 ng/mL in the serum‐containing medium for 7 days. For restimulation experiment, naïve CD4^+^ T cells differentiated with 10 ng/mL TGF‐β1 (Cell Signaling Technology) were collected on day 7 and washed and incubated without TCR stimulation or cytokines for 2 days. On day 9, cells were restimulated with anti‐CD3/28 antibodies at the same concentration with the first stimulation and cultured in the absence or presence of IL‐1β (Wako), TNF‐α (Miltenyi), IL‐6 (Wako; each 10 ng/mL), and/or TGF‐β1 (10 ng/mL) for 4 days. A TGF‐β signaling inhibitor, SB431542 and a SMAD3 inhibitor, SIS3 were purchased from Stemgen and Cayman, respectively. For the IL‐2‐neutralization experiments, naïve CD4^+^ T cells were cultured with 10 ng/mL TGF‐β1, and plate‐bound 5 μg/mL anti‐CD3 and 10 μg/mL anti‐CD28 antibodies in the presence of neutralizing anti‐IL‐2 antibody.

### Antibodies for flow cytometry and cell sorting

Brilliant Violet 421‐conjugated anti‐CD3 (UCTH1), PerCP‐Cy5.5‐conjugated anti‐CD4 (RPA‐T4), anti‐CD25 (M‐A251), anti‐CXCR5 (J252D4), and control mouse IgG1, FITC‐conjugated anti‐CD45RA (H100), APC‐conjugated anti‐CD4 (KAT‐4), and control mouse IgG1 and IgG2b, PE‐Cy7‐conjugated anti‐CTLA4 (L3D10), and control mouse IgG1, and APC‐Cy7‐conjugated anti‐PD‐1 (EH12.2.H7) and control mouse IgG1 were obtained from BioLegend. FITC‐conjugated anti‐GITR (eBioAITR) and control mouse IgG1, PE‐conjugated anti‐FoxP3 (236A/E7), anti‐IL‐21 (3A3‐N2), and control mouse IgG1, PE‐Cy7‐conjugated anti‐ICOS (ISA‐3) were obtained from eBioscience. PE‐conjugated and anti‐STAT5A (251610), and APC‐conjugated anti‐CXCL13 (53610) and anti‐STAT5B (389215) were obtained from R&D Systems. FITC‐conjugated anti‐CD19 (HIB19) and PE‐conjugated anti‐BCL6 (K112‐91) was obtained from BD Bioscience.

### Flow cytometry

For intracellular staining, cells were cultured for 5 h with 4 μM monensin (Sigma‐Aldrich) in the absence of PMA (Nacalai Tesque) or ionomycin (Nacalai Tesque) to prevent unexpected modification of gene expression. Only when intracellular IL‐21 was stained, cells were cultured for 5 h with 4 μM monensin, 10 ng/mL PMA, and 1 μM ionomycin. After staining of surface molecules, intracellular molecules were stained using Foxp3/Transcription Factor Staining Buffer Set (eBioscience). For the intracellular staining of STAT5A and STAT5B, cells were fixed with Cytofix™ Fixation Buffer (BD bioscience) and permeabilized with Perm Buffer III (BD bioscience). The data were acquired with a FACSCanto II flow cytometer (BD Biosciences) and were analyzed with FlowJo Version 10 (Tree Star). Fixable Viability Dye eFluor 506 (eBioscience) was used to exclude dead cells. The border of gates was determined according to the staining with isotype controls.

### ELISA

The concentration of CXCL13 in the supernatant was measured with Flex Station 3 (Molecular Devices) using Human CXCL13/BLC/BCA‐1 DuoSet (R&D systems) with a detection limit of 8 pg/mL.

### Migration assay

Supernatant of naïve CD4^+^ T cells differentiated with 10 ng/mL TGF‐β1 for 7 days was harvested and frozen until analyses. One million fresh PBMCs of healthy volunteers were loaded in IMDM containing 1.75% human serum of the upper well and cultured for 4 h. Cells migrating into IMDM containing 50% of the supernatant in the presence of the indicated antibody at the concentration of 15 μg/mL were analyzed by flow cytometry.

### Quantitative PCR

GFP‐positive cells transduced with control or STAT5‐specific shRNA were sorted with FACSAria II (BD Biosciences). Dead cells were excluded with 7‐amino‐actinomycin D (Sigma Aldrich). mRNA extraction and cDNA synthesis were performed using SuperPrep® Cell Lysis & RT Kit for qPCR (TOYOBO).

Tonsil CD3^+^CD4^+^CXCR5^hi^ICOS^hi^ Tfh cells were sorted with FACSAria II followed by fixation with Foxp3/Transcription Factor Fixation/Permeabilization Buffer (eBioscience) to obtain mRNA with same protocol with CXCL13^+^CD4^+^ cells. CXCL13^+^CD4^+^ T cells were sorted from naïve CD4^+^ T cells differentiated in the presence of TGF‐β1, following cell fixation and intracellular staining of CXCL13. mRNA was extracted using RNeasy FFPE kit (Qiagen). Reverse transcription was performed using a SuperScript® VILO cDNA Synthesis Kit (Life technologies).

qRT‐PCR was performed using THUNDERBIRD™ SYBR® qPCR Mix (TOYOBO) on LightCycler®480 (Roche Applied Science) with the following primers: CXCL13 (5′‐TCTCTGCTTCTCATGCTGCT‐3′ and 5′‐TCAAGCTTGTGTAATAGACCTCCA‐3′), STAT3 (5′‐CCCTTGGATTGAGAGTCAAGA‐3′ and 5′‐AAGCGGCTATACTGCTGGTC‐3′), SH2D1A (5′‐AGGCGTGTACTGCCTATGTG‐3′ and 5′‐GTACCCCAGGTGCTGTCTCA‐3), CD40LG (5′‐TCATGAAAACGATACAGAGATGC‐3′ and 5′‐CTTCGTCTCCTCTTTGTTTAACATT‐3′), ICOS (5′‐GGATGCATACTTATTTGTTGGCTTA‐3′ and 5′‐TGTATTCACCGTTAGGGTCGT‐3′), CXCR5 (5′‐GCCATGAACTACCCGCTAAC‐3′ and 5′‐TCTGTCCAGTTCCCAGAACA‐3′), CD200 (5′‐AGGATGGAGAGGCTGGTGA‐3′ and 5′‐ACCACTGCTGCCATGACC‐3′), IL21 (5′‐AGGAAACCACCTTCCACAAA‐3′ and 5′‐GAATCACATGAAGGGCATGTT‐3′), TIGIT (5′‐GCTGGTGTCTCCTCCTGATCT‐3′ and 5‐TGTGCCTGTCATCATTCCTG‐3′), STAT5A (5′‐TCCCTATAACATGTACCCACAGAA‐3′ and 5′‐ATGGTCTCATCCAGGTCGAA‐3′), STAT5B (5′‐GAAGATCAAGCTGGGGCACT‐3′ and 5′‐CATGGCATCAGCAAGGCTTC‐3′), and GAPDH (5′‐CGCTCTCTGCTCCTCCTGTT‐3′ and 5′‐CCATGGTGTCTGAGCGATGT‐3′). Alternatively, predesigned TaqMan Gene Expression Assays (Life technologies; BCL6 Hs00153368_m1, MAF Hs04185012_s1, and BATF Hs00232390_m1) was performed with LightCycler® TaqMan® Master (Roche Applied Science) on LightCycler®480. The expression of mRNA was normalized by that of GAPDH. For the heat map presentation, the average of log mRNA expression for each gene was defined as 1.

### Lentivirus production and transduction

The cDNA encoding human FoxP3 was cloned from that of human PBMC, and inserted into multicloning sites of CSII‐EF‐MCS‐IRES2‐Venus (provided by Dr. H. Miyoshi, RIKEN BioResource Center), expressing a variant of yellow fluorescent protein.

For the shRNA lentivirus, synthesized oligonucleotides were inserted into pENTR/U6 vector and subsequently transferred to the lentiviral destination vector CS‐RfA‐EG (RIKEN BioResource Center), a lentiviral vector expressing GFP with a Gateway destination cassette, by performing an LR clonase reaction (Life Sciences). Target sequences and hairpin sequences for shRNA were as follows: for human FoxP3, 5′‐GCATGTTTGCCTTCTTCAGAA‐3′ and 5′‐AGAGCTTG‐3′ 49; for both STAT5A and STAT5B, 5′‐GCAGAAACTGTTCAACAACAG‐3′ and 5′‐CTCGAG‐3′; and negative control, 5′‐ATCCGCGCGATAGTACGTATT‐3′ and 5′‐CTCGAG‐3′.

HEK293T cells were transiently transfected with expression vector, packaging plasmid (pCAG‐HIVgp), and expression plasmid for vesicular stomatitis virus G glycoprotein and Rev (pCMV‐VSV‐G‐RSV‐Rev). Lentiviral supernatants were collected 72 h after transfection and concentrated by ultracentrifugation at 36000 × *g* for 2 h. Naïve CD4^+^ T cells were stimulated with anti‐CD3/28 antibodies for 24 h without cytokines, transduced with lentiviral supernatant at a multiplicity of infection of 10–50 by 90 min centrifugation of 3200 × *g* at 32°C. Cytokines were added just after the transduction for overexpression, and 18 h after transduction for shRNA, followed by flow cytometry on day 7.

### Statistical analysis

The data were analyzed using two‐tailed Student's *t*‐test or paired Student's *t*‐test as appropriate. A *p*‐value < 0.05 was considered significant.

## Conflict of interest

Astellas Pharma had no role in the study design or in the collection, analysis, or interpretation of the data; the writing of the manuscript; or the decision to submit the manuscript for publication. Publication of this article was approved by an intellectual property committee composed of representatives from Kyoto University and Astellas Pharma. Raw data cannot be provided due to confidentiality agreements.

**Abbreviations**
BCL6: B‐cell lymphoma 6
BMP: bone morphogenetic protein
CTLA4: cytotoxic T‐lymphocyte‐associated antigen 4
ELS: ectopic lymphoid‐like structure
GITR: glucocorticoid‐induced TNF receptor‐regulated protein
iTreg: induced Treg
PD‐1: programmed death 1
RA: rheumatoid arthritis
Tfh: follicular helper T

## Supporting information

As a service to our authors and readers, this journal provides supporting information supplied by the authors. Such materials are peer reviewed and may be re‐organized for online delivery, but are not copy‐edited or typeset. Technical support issues arising from supporting information (other than missing files) should be addressed to the authors.

Supporting Information
