# Supplementary material for: TGF‐β induces the differentiation of human CXCL13‐producing CD4+ T cells
Source: Eur J Immunol. 2015 Nov 24;46(2):360–71. doi: 10.1002/eji.201546043 (PMC5063156; doi:10.1002/eji.201546043)
Supplement: Supplementary file 1 — Supporting Information [file EJI-46-360-s001.pdf]

Supporting Information for

TGF-β induces the differentiation of human CXCL13-producing $CD4^+$ T cells

Shio Kobayashi[1], Takeshi Watanabe[2], Ryo Suzuki[3], Moritoshi Furu[4], Hiromu Ito[5], Juichi Ito[3], Shuichi Matsuda[5], Hiroyuki Yoshitomi[1,5,6] *

[1] Center for Innovation in Immunoregulative Technology and Therapeutics, Kyoto University Graduate School of Medicine, Kyoto, Japan

[2] The Tazuke-Kofukai Medical Research Institute / Kitano Hospital, Osaka, Japan

[3] Department of Otolaryngology, Head and Neck Surgery, Kyoto University Graduate School of Medicine, Kyoto, Japan

[4] Department of the Control for Rheumatic Diseases, Kyoto University Graduate School of Medicine, Kyoto, Japan

[5] Department of Orthopaedic Surgery, Kyoto University Graduate School of Medicine, Kyoto, Japan

[6] Department of Tissue Regeneration, Institute for Frontier Medical Sciences, Kyoto University, Kyoto Japan

*Correspondence: yositomi@kuhp.kyoto-u.ac.jp

Supporting Information

Materials and Methods relating Supporting Information Figure 1, 2, 3, and 6.

Cell culture

Cells were cultured in a humidified, 5% CO_2 incubator at 37°C with IMDM (Life Technologies) supplemented with 3.5% human AB serum (Lonza), and 100 unit/ml penicillin and streptomycin (Life Technologies). Blood $CD4^+$ T cells were stimulated and cultured in each Th condition. For Th0 differentiation, cells were stimulated with plate-bound 1 μg/ml anti-CD3 (OKT3, eBioscience) and soluble 10 μg/ml anti-CD28 (CD28.2, eBioscience) antibodies and cultured in the presence of 5 μg/ml anti-IFN-γ (NIB42, BioLegend) and 5 μg/ml anti-IL-4 (MP4-25D2, BioLegend) antibodies without cytokines; For Th1 differentiation, 2.5 μg/ml anti-CD3, 2.5μg/ml anti-CD28, and 5 μg/ml anti-IL-4 antibodies and 10 ng/ml IL-12 (Peprotech); For Th2 differentiation, 1 μg/ml anti-CD3, 10 μg/ml anti-CD28, and 5 μg/ml anti-IFN-γ antibodies and 10 ng/ml IL-4 (Peprotech); For Th17 differentiation, 1 μg/ml anti-CD3, 10 μg/ml anti-CD28, 5 μg/ml anti-IFN-γ, and 5 μg/ml anti-IL-4 antibodies and IL-1β (Miltenyi) , IL-6 (Wako) and TGF-β1 (Cell Signaling Technology; each 10 ng/ml). TGF-β2 and TGF-β3 or BMP2 and BMP4 were purchased from Cell Signaling Technology or R&D systems.

Intracellular staining

When intracellular cytokines were stained with FITC-conjugated anti-IFN-γ (B27, BD Bioscience), PE-conjugated anti-IL-4 (8D4-8, Biolegend), and PE-Cy7-conjugated anti-IL-17 (BL168, Biolegend), cells were cultured for 5 h with 4 μM monensin, 10 ng/ml PMA and 1 μM ionomycin. Intracellular molecules were stained using Foxp3/Transcription Factor Staining Buffer Set (eBioscience). Fixable Viability Dye eFluor 506 (eBioscience) was used to exclude dead cells. The border of gates was determined according to the staining with isotype controls.

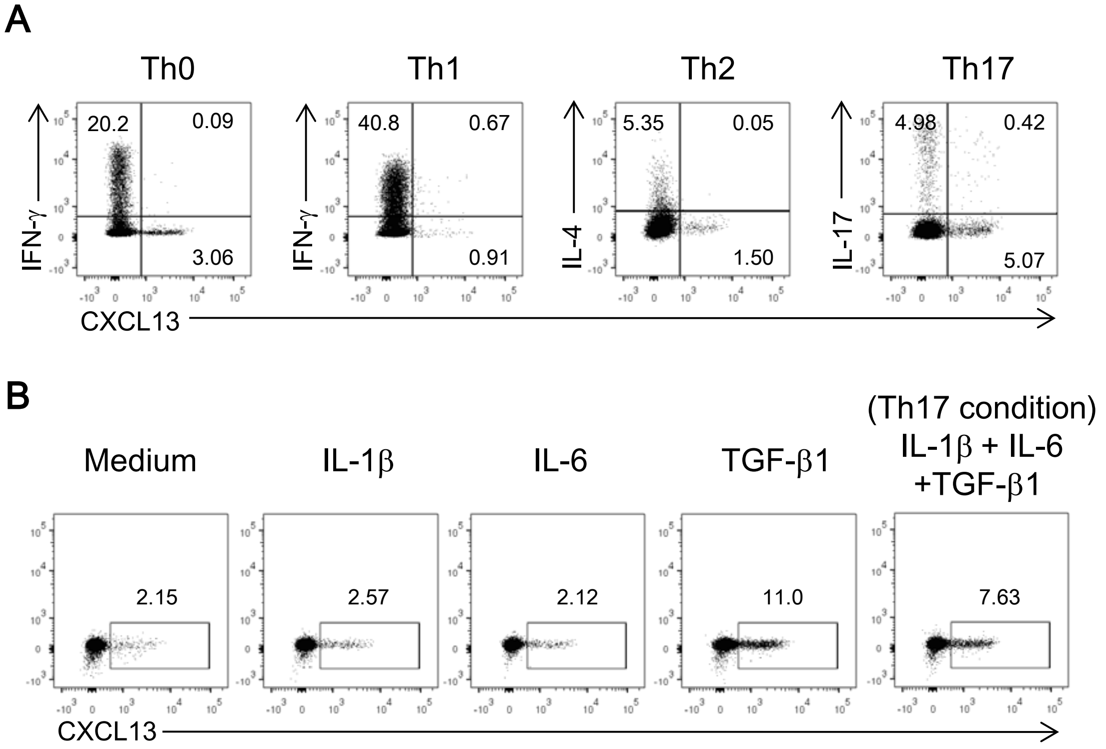

Supporting Information Figure 1. Differentiation of blood CD4$^+$ T cells with TGF-β1 induces CXCL13-producing CD4$^+$ T cells. (A) Flow cytometry of human blood CD4$^+$ T cells cultured under the Th0, Th1 (with IL-12), Th2 (with IL-4), or Th17 (with IL-1β, IL-6, and TGF-β1) conditions for 7 days, following the activation with PMA/ionomycin. Please see supporting information materials and methods for the detailed culture conditions. (B) Human blood CD4$^+$ T cells were activated with 1 μg/ml anti-CD3 and 10 μg/ml anti-CD28 (same with Th17 conditions) in the presence of each cytokine contained in the Th17-polarizing cocktail, followed by intracellular staning without PMA/ionomycin. Percentages of CXCL13$^+$ cells were determined with flow cytometry. The gates were determined according to the staining with isotype controls. Numbers in plots indicate the percentage of cells in each area. Data represent at least three independent experiments.

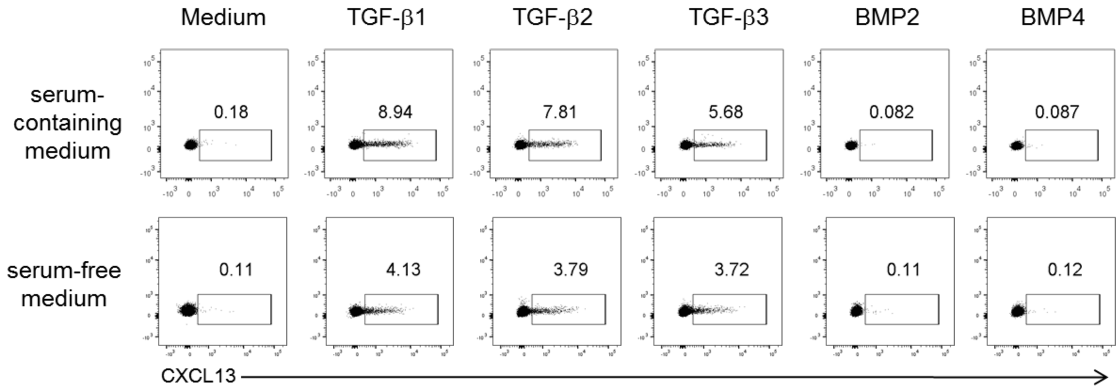

Supporting Information Figure 2. Influence of TGF-β and BMP signaling on the differentiation of CXCL13-producing cells. Human naïve blood $CD4^+$ T cells were cultured with anti-CD3/28 antibodies in the presence of the indicated TGF-β or BMP at the concentration of 10 ng/ml in serum-containing medium or in serum-free medium for 7 days. Percentages of $CXCL13^+$ cells were determined with flow cytometry. The gates were determined according to the staining with isotype controls. Numbers in plots indicate the percentage of cells in each area. Data represent at least three independent experiments.

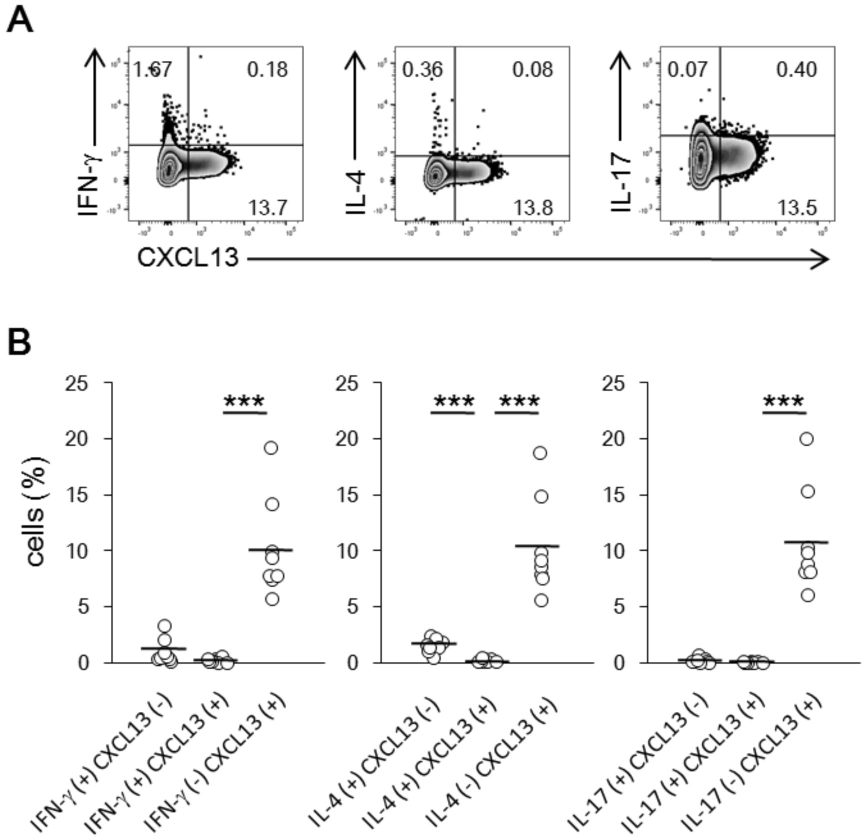

Supporting Information Figure 3. Th cytokine production by TGF-β-induced CXCL13-producing CD4$^+$ T cells. (A and B) Intracellular staining of cytokines and CXCL13 in naïve CD4$^+$ T cells of eight healthy volunteers differentiated with TGF-β1, following PMA/ionomycin treatment. (A) Representative dot plots, and (B) summaries of indicated populations were determined with flow cytometry. The border of the quadrants was determined according to the staining with isotype controls. Numbers in plots indicate the percentage of cells in each area. Each symbol represents an individual sample and bars represent means. ***p<0.01, paired Student's *t* test.

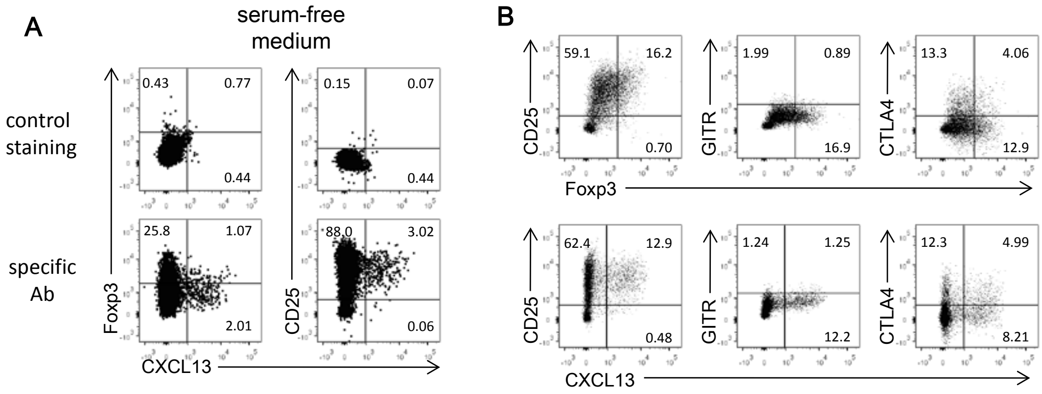

Supporting Information Figure 4. Expression of Treg markers in TGF-β-induced CXCL13-producing CD4$^+$ T cells differentiated in serum-free medium. (A and B) Naïve CD4$^+$ T cells were differentiated with TGF-β1 in serum-free medium. (A) The expression of FoxP3 and CD25 in CXCL13$^+$ cells and (B) the expression of CD25, GITR and CTLA4 in FoxP3$^+$ cells or CXCL13$^+$ cellls were determined with flow cytometry. The border of the quadrant was determined according to the staining with isotype controls. Data represent eight independent experiments.

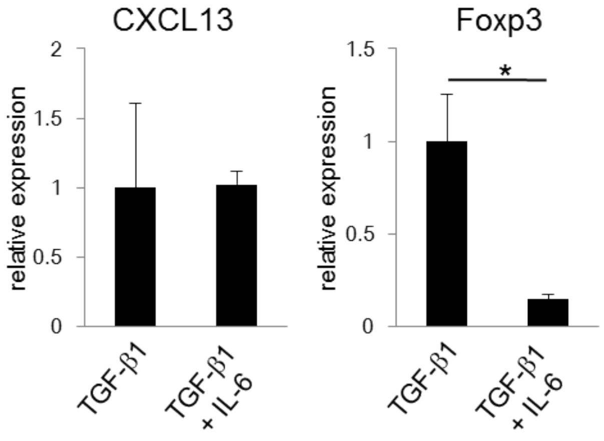

Supporting Information Figure 5. Influence of IL-6 on the induction of Foxp3 and CXCL13. Human naïve blood $CD4^+$ T cells were cultured with 10 ng/ml TGF-β1 in the presence or absence of 10 ng/ml IL-6 for 7 days. The ratio of frequencies of $CXCL13^+$ or $Foxp3^{hi}$ cells among cells cultured with TGF-β1 and IL-6 to those with TGF-β1 alone was determined with flow cytometry. Data are shown as mean + SD of triplicates from a single experiment representative of three experiments performed. $^*p<0.05$, two-tailed Student's *t* test.

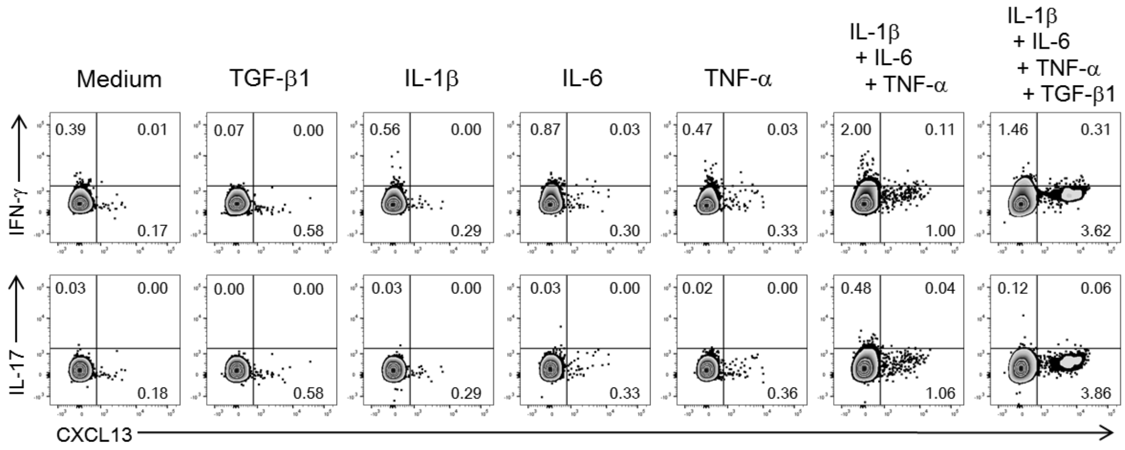

Supporting Information Figure 6. Production of IFN-γ and IL-17 by TGF-β-induced CXCL13-producing $CD4^+$ T cells restimulated with TCR under several cytokine conditions. Naïve $CD4^+$ T cells were activated in the presence of TGF-β1 for 7 days, rested in basal medium for 2 days, and restimulated with anti-CD3/28 antibodies in the presence of TGF-β1, IL-1β, IL-6, and/or TNF-α for 4 days, followed by intracellular staining after 5 h activation with PMA/ionomycin as in figure 5C-E. The expression of IFN-γ, IL-17 and CXCL13 was determined with flow cytometry. The border of the quadrant was determined according to the staining with isotype controls. Numbers in plots indicate the percentage of cells in each area. Data represent at least three independent experiments.
